# A case of pancreaticoduodenal artery bleeding after laparoscopic right colectomy requiring open hemostasis

**DOI:** 10.1186/s40792-024-01816-x

**Published:** 2024-01-16

**Authors:** Rika Ono, Tetsuro Tominaga, Takashi Nonaka, Yuma Takamura, Kaido Oishi, Toshio Shiraishi, Shintaro Hashimoto, Keisuke Noda, Terumitsu Sawai, Takeshi Nagayasu

**Affiliations:** Department of Surgical Oncology, Nagasaki University Graduate School of Biomedical Science, 1-7-1 Sakamoto, Nagasaki, 852-8501 Japan

**Keywords:** Bleeding, Pancreaticoduodenal artery, Right colectomy

## Abstract

**Background:**

Pancreatic and duodenal-related complications after right colectomy carry a higher risk of mortality.

**Case presentation:**

A 64-year-old woman underwent laparoscopic right colectomy for a laterally spreading tumor in the cecum. On postoperative day 10, she experienced sudden hematemesis. Contrast-enhanced computed tomography (CT) of the abdomen showed a large amount of hemorrhage in the stomach, but no obvious extravasation. In addition, free air was observed near the duodenal bulb. Despite blood transfusion, vital signs remained unstable and emergency surgery was performed. The abdomen was opened through midline incisions in the upper and lower abdomen. A fragile wall and perforation were observed at the border of the left side of the duodenal bulb and pancreas, with active bleeding observed from inside. As visualization of the bleeding point proved difficult, the duodenum was divided circumferentially to confirm the bleeding point and hemostasis was performed using 4-0 PDS. The left posterior wall of the duodenum was missing, exposing the pancreatic head. For reconstruction, the jejunum was elevated via the posterior colonic route and the duodenal segment and elevated jejunum were anastomosed in an end-to-side manner. Subsequently, gastrojejunal and Brown anastomoses were added. Drains were placed before and after the duodenojejunal anastomosis. Postoperative vital signs were stable and the patient was extubated on postoperative day 1. Follow-up contrast-enhanced CT of the abdomen showed no active bleeding, and the patient was discharged home on postoperative day 21. As of 6 months postoperatively, the course of recovery has been uneventful.

**Conclusions:**

We encountered a case of pancreaticoduodenal artery hemorrhage after laparoscopic right colectomy. Bleeding at this site can prove fatal, so treatment plans should be formulated according to the urgency of the situation.

## Background

Postoperative hemorrhage from colorectal cancer is a relatively rare complication, with a reported incidence of 0.26% among patients with colorectal cancer in 2021 in Japan [1–3]. However, this serious complication can lead to sudden circulatory changes and death. In addition, the Japanese National Clinical Database (NCD) has shown that right colon resection has a lower complication rate but a higher mortality rate than left peripheral colon resection [4]. In general, pancreatic and duodenal-related complications are considered more likely to be serious, and right colon resection carries a higher risk of fatal complications because the surgical manipulation required around that site is more extensive [5].

In this report, we describe a case of pancreaticoduodenal artery hemorrhage on day 10 after right colon resection, which was successfully treated by emergency laparotomy.

## Case presentation

**Fig. 1:**
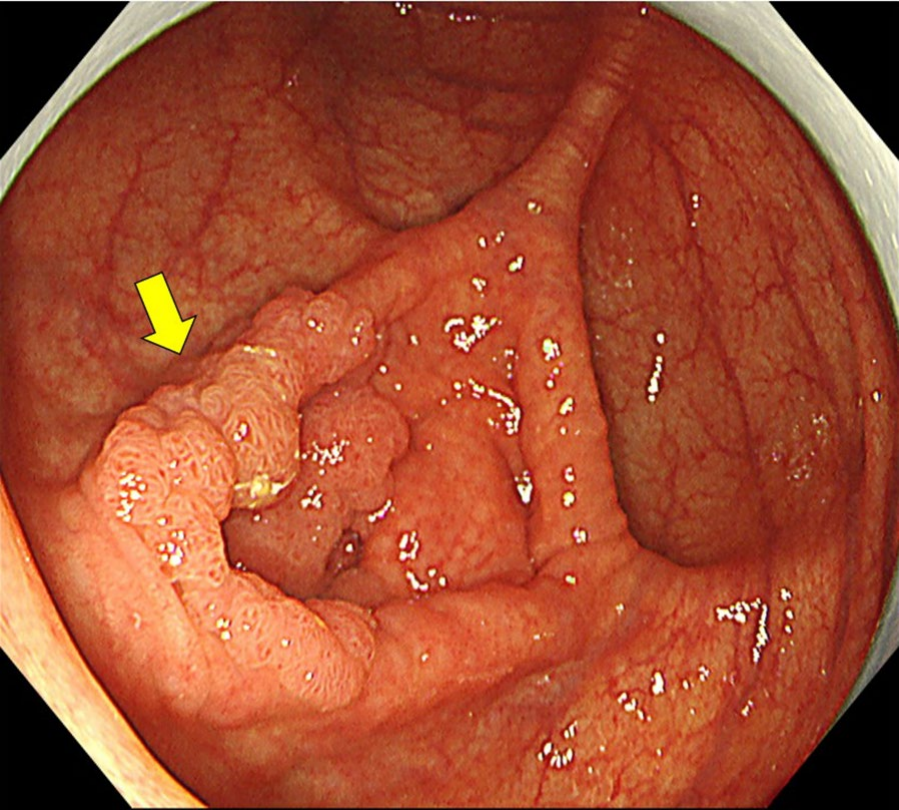
Colonoscopy. Colonoscopy shows a 30 mm, laterally spreading tumor located in the cecum

**Fig. 2:**
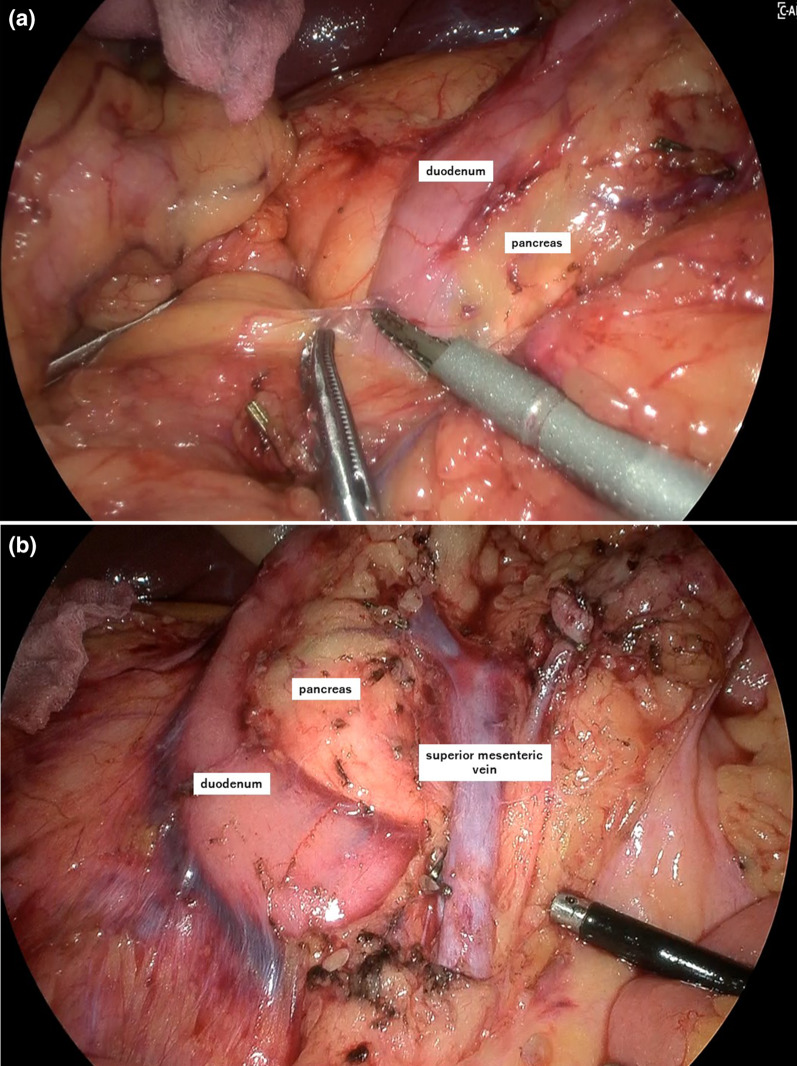
Intraoperative findings. **a**, **b** Dissection between the mesentery and duodenum (**a**) followed by lymph node dissection (**b**)

**Fig. 3:**
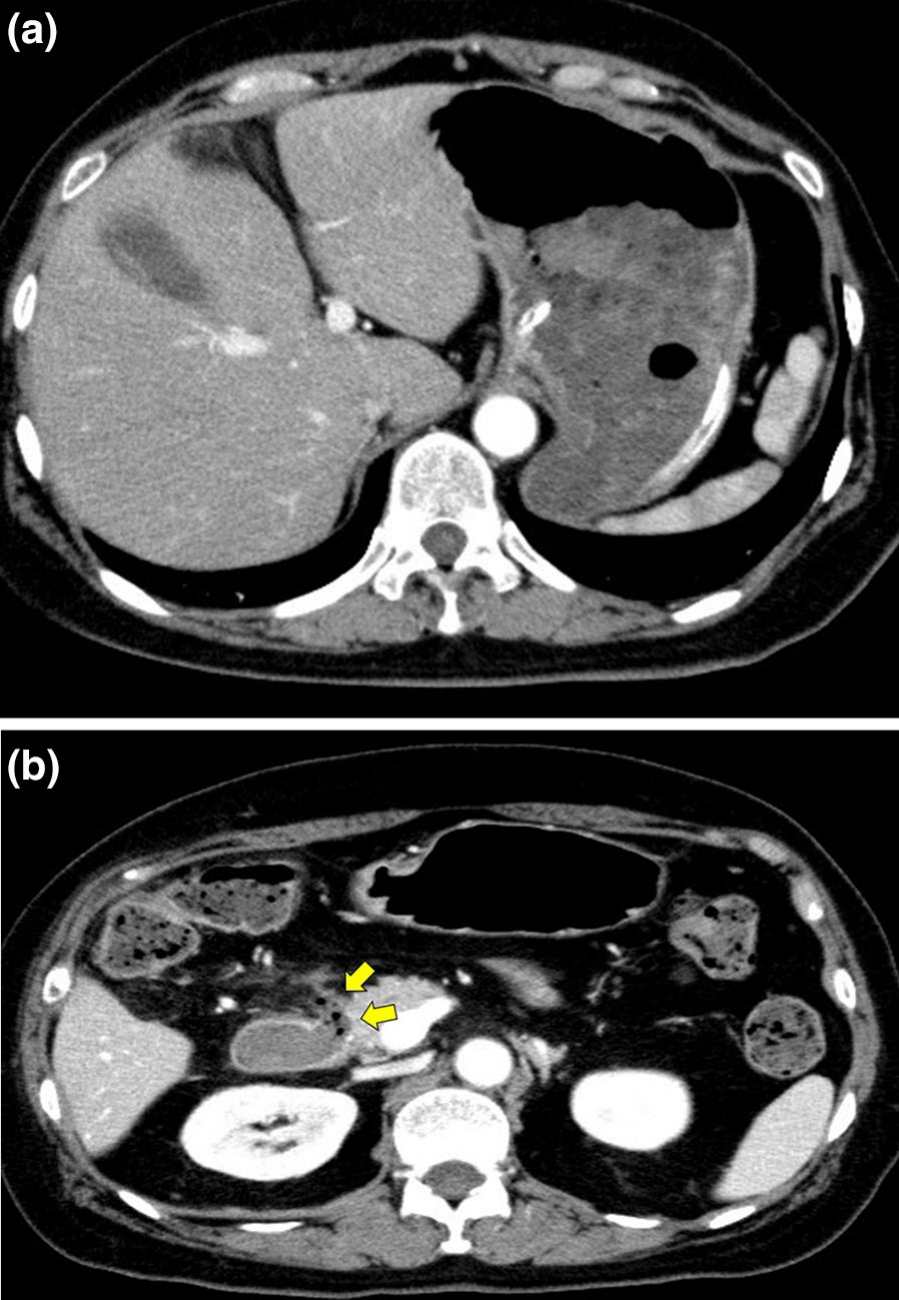
Contrast-enhanced CT. **a** Contrast-enhanced CT of the abdomen shows a large amount of hemorrhage in the stomach, suggesting massive gastrointestinal bleeding. **b** Free air is observed near the duodenal bulb (arrow)

**Fig. 4:**
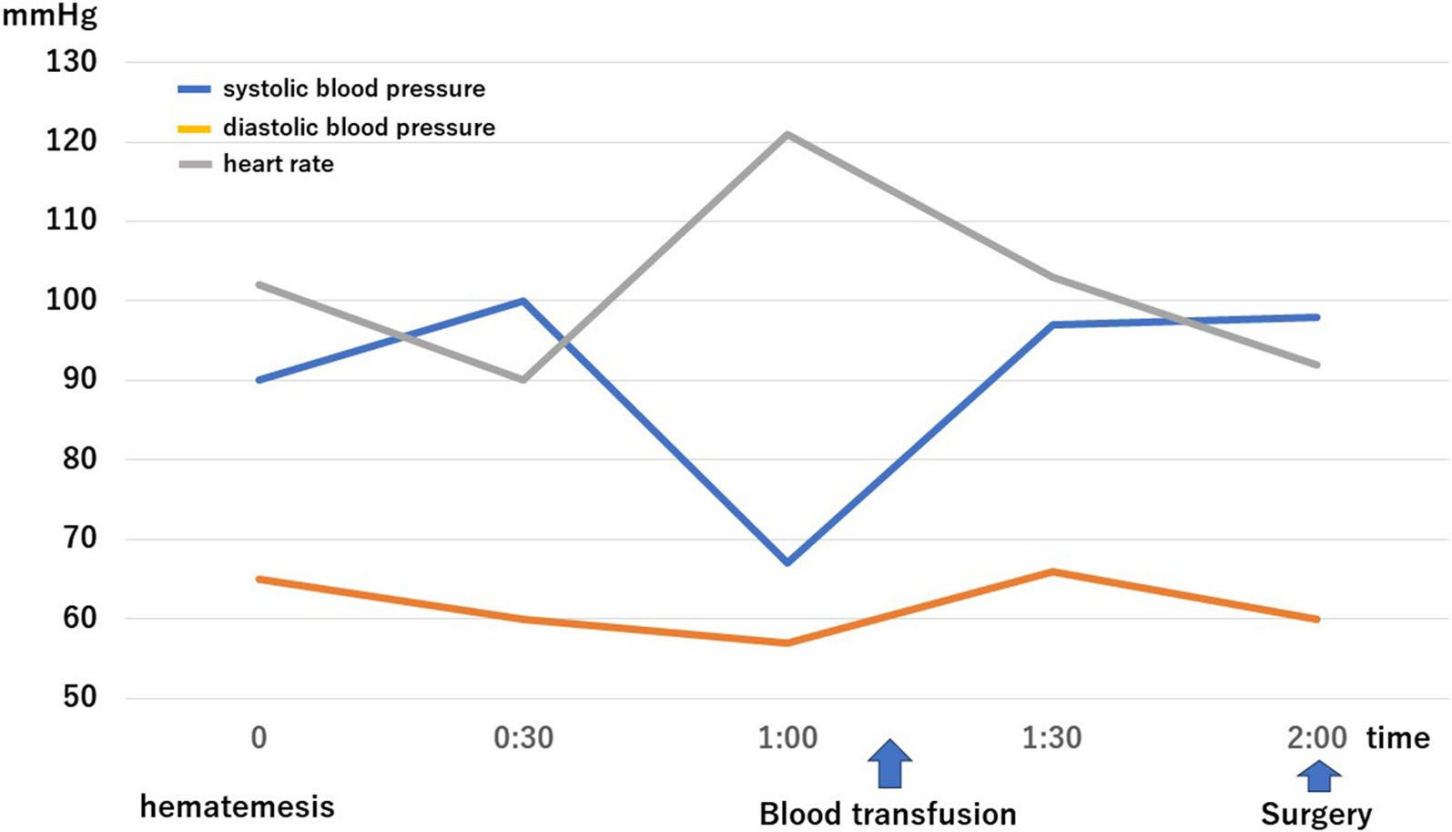
Course of gastrointestinal hemorrhage

**Fig. 5:**
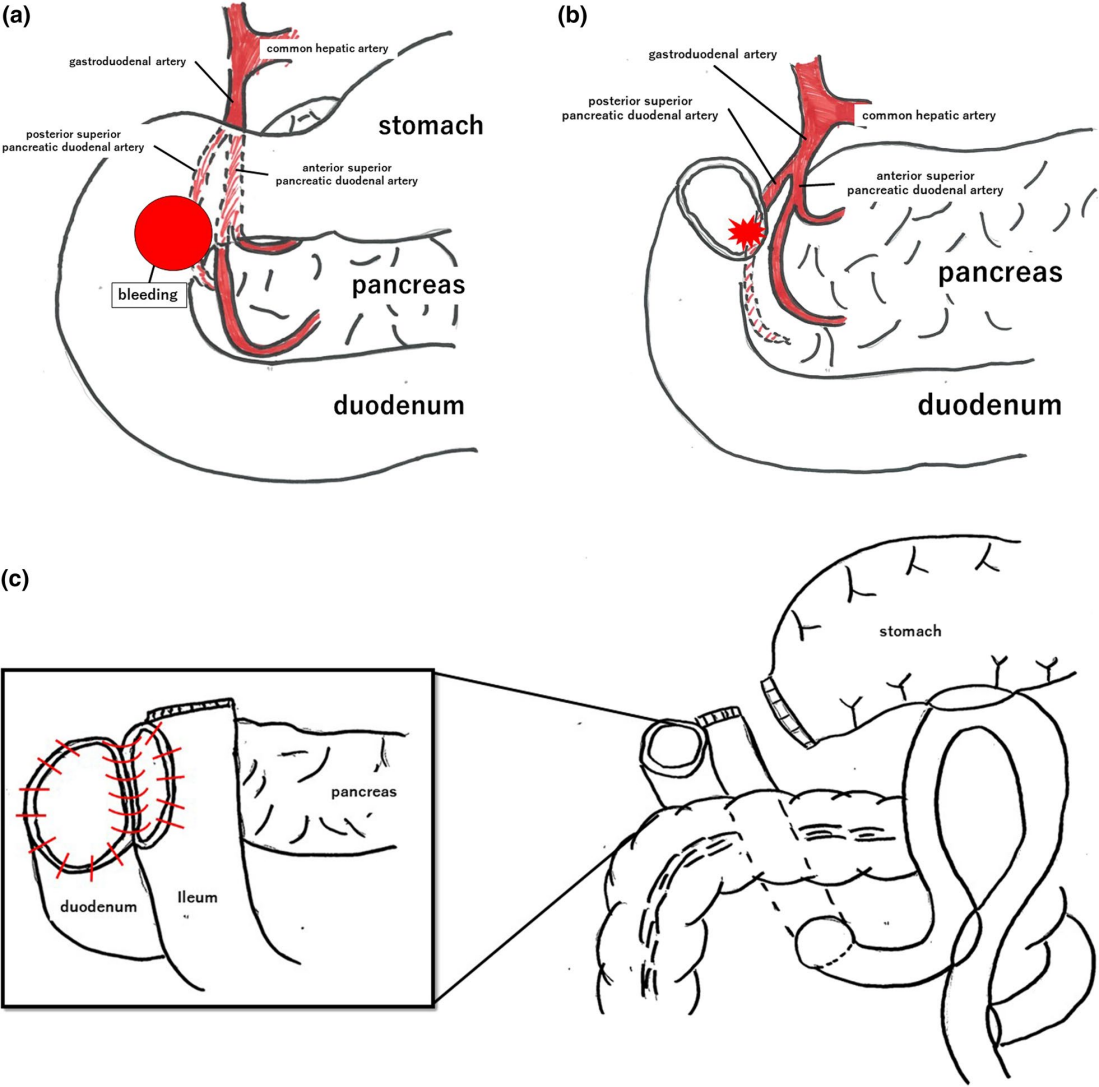
Schema of emergency surgery. **a** Duodenal bulb is partially perforated with active bleeding from the inside. **b** After dissection of the duodenum. **c** Reconstruction. The jejunum is dissected and elevated via the posterior colonic route. The ascending jejunum and transected duodenum are anastomosed at the end, with the addition of gastric jejunal anastomosis and Brown anastomosis

A 64-year-old woman had undergone lower gastrointestinal endoscopy by her previous physician after fecal occult blood testing during a medical checkup yielded a positive result. The patient was referred to our department for surgery after endoscopic examination revealed a lesion in the ileocecal region with substantial extension into the appendiceal orifice, and endoscopic treatment was judged too difficult. The patient was 152 cm tall, weighed 57 kg, and had a body mass index of 24.5 kg/m^2^. She had a history of chronic renal failure and lipid metabolism disorder. Preoperative blood samples showed no anemia and no elevation of tumor marker levels. Lower gastrointestinal endoscopy revealed a 30 mm, laterally spreading tumor in the cecum, occupying two-thirds of the appendiceal opening (Fig. 1). A biopsy specimen revealed a suspected well-differentiated adenocarcinoma. Thoracoabdominal computed tomography (CT) showed no obvious regional lymphadenopathy or distant metastasis. The preoperative diagnosis was appendiceal cancer cT1N0M0 stage I. We planned laparoscopic right colectomy plus Japanese D3 lymph node dissection. The surgery was performed through 5 ports. A camera port was inserted at the umbilicus and forceps ports were inserted in a square pattern to the left and right upper and lower abdomen. Starting with a cranial approach, the mesenteric base of the transverse colon was identified with the pancreatic submargin and dissection was advanced between the stomach and duodenum. Adhesion between the transverse colon and duodenum was strong, and each piece was carefully dissected and the hepatic curvature was taken down. No damage requiring repair was evident in the duodenum within visible range (Fig. 2a, b). The ileocecal vein/artery and right branch of the middle colonic artery were clipped and resected. After mesenteric processing, the specimen was dissected with a stapler and removed. Reconstruction was performed intracorporeally by delta anastomosis. The operation time was 2 h 52 min, with blood loss of 7 ml. The pathological diagnosis was high-grade tubular adenoma. Postoperatively, the patient developed temporary ileus and required nasogastric intubation, but she recovered without further intervention. Postoperative laboratory data showed slight anemia (hemoglobin 10.7 g/dl) and slightly elevated C-reactive protein (10.6 g/l), with gradual improvements over time. The patient was scheduled for discharge from hospital on postoperative day 10, but on the morning of the proposed day of discharge, she suddenly vomited blood. Contrast-enhanced CT of the abdomen showed a large amount of hemorrhage in the stomach, suggesting a large volume of gastrointestinal bleeding (Fig. 3a). Free air was observed near the duodenal bulb (Fig. 3b). No extravasation was apparent. Despite urgent blood transfusion, vital signs remained unstable and emergency surgery was performed for upper gastrointestinal bleeding and suspected perforation of the upper gastrointestinal tract (Fig. 4). When the laparotomy was performed through upper and lower midline abdominal incisions, pale hematogenous ascites was observed around the duodenum. The left side of the duodenal bulb, bordering the pancreas, showed an area of fragile duodenal wall and partial perforation, with active bleeding from the inside (Fig. 5a). However, visualizing the origin of bleeding in the duodenum proved difficult, so the duodenum was divided circumferentially to confirm the bleeding point, then hemostasis was achieved with ligation (Fig. 5b). The left posterior wall of the duodenum was found to be missing, exposing the pancreas. For reconstruction, the jejunum was separated with a stapler then elevated by the posterior colonic route. The elevated jejunum and duodenal section were then anastomosed in an end-to-side manner. The gastric transection was detached with a stapler after trimming. Gastric jejunal anastomosis and Brown anastomosis were added (Fig. 5c). A drain was placed before and after the duodenojejunal anastomosis, and the surgery was completed. The operation time was 3 h 25 min, with blood loss of 1351 ml. Postoperatively, the patient was admitted to an intensive care unit for systemic management. Postoperative vital signs were stable and she was extubated on postoperative day 1 and transferred to a general ward on postoperative day 3. Follow-up CT of the abdomen on postoperative day 5 showed no active bleeding, and the patient started drinking water on postoperative day 7. She resumed eating on postoperative day 10. As abdominal symptoms did not worsen, she was discharged home on postoperative day 21.

## Discussion

Postoperative bleeding after colorectal cancer surgery is a relatively rare complication in Japan, with an incidence of 0.26% (98/36,633) based on 2021 data, but the condition can cause rapid circulatory changes and is potentially fatal [1–3]. In addition, NCD analysis suggests that while right hemicolectomy is associated with a lower incidence of postoperative use of an eight-part garment compared to low anterior rectal resection (7.4% vs 11.9%, respectively), the 30-day mortality rate is higher (1.2% vs 0.4%, respectively) and those complications that do develop tend to be more severe [4]. These findings suggest that because right hemicolectomy often involves manipulation of the pancreatic head and peri-duodenal area during surgical maneuvers, the extra manipulation involved increases the risks of pancreatic leakage and duodenal injury [5]. In the present case, extensive dissection of the border between the mesentery and duodenum/pancreatic head was performed during laparoscopic right colectomy, resulting in bleeding from the pancreaticoduodenal artery.

The cause of this hemorrhage could have been: (1) bleeding due to perforation of a duodenal ulcer; or (2) formation of a small pseudoaneurysm due to intraoperative injury or spillover inflammation such as from leaked pancreatic juice [6, 7]. Regarding the first option, the patient was under high physical stress from the major surgery and the lesion was located near the duodenal bulb, where ulcers tend to occur, consistent with the clinical course observed. On the other hand, the facts that the patient had been taking a proton-pump inhibitor before surgery and that a stress ulcer developed on postoperative day 10, whereas stress ulcers tend to develop in the early postoperative period, are inconsistent with the clinical findings. Regarding the possibility of intraoperative injury, no obvious thermal or physical injury requiring repair was observed on review of the intraoperative video. Although no evidence of prolonged inflammation was apparent during the postoperative period, the possibility of localized inflammation cannot be ruled out. In this case, no obvious cause of bleeding was identified. However, potentially fatal complications can arise following right colectomy, as in this case, although the frequency is low. The response to each risk factor needs to be considered.

Treatments for gastrointestinal bleeding include endoscopic hemostasis, interventional radiology (IVR), and surgery [8]. Endoscopic hemostasis is less invasive, while IVR has the advantage of being able to pinpoint the bleeding point and stop the bleeding with contrast medium. However, if the bleeding point cannot be identified, hemostasis can be difficult to achieve. Surgery requires general anesthesia and is more invasive to the patient, but has the advantage of allowing direct identification of the bleeding origin and rapid control of the bleeding. In this case, preoperative contrast-enhanced CT showed a large amount of hemorrhage in the stomach, and endoscopic hemostasis was expected to prove difficult to achieve. In addition, because no obvious extravasation was noted, there was a possibility that IVR hemostasis would prove inadequate. Furthermore, the vital signs of the patient remained unstable even after blood transfusions and other measures, so emergency laparotomy was selected to ensure rapid and reliable hemostasis. As a result, we were able to immediately identify the bleeding point and stop the bleeding, which we believe saved the patient's life.

The literature on pancreaticoduodenal hemorrhage remains limited, with most reports involving pancreaticoduodenal aneurysms [9–12]. Stanley et al. reported that abdominal visceral aneurysms are rare, accounting for 0.1–0.2% of all aneurysms, among which pancreaticoduodenal aneurysms are even rarer, accounting for about 2% of abdominal visceral aneurysms [12]. The risk of aneurysm rupture is as high as 50%, and the mortality rate is as high as 50%, indicating that this is a high-risk location. IVR and surgery are recommended as treatments, but since the aneurysm is controlled by two arteries (the celiac and superior mesenteric arteries), embolization of only one may result in inadequate hemostasis, and about 15% of cases are difficult to treat with IVR. In cases requiring rapid hemostasis, treatment strategies should be considered with care.

## Conclusions

We encountered a case of pancreaticoduodenal artery hemorrhage after laparoscopic right colectomy. Bleeding at this site can prove fatal, and a treatment plan should be formulated according to the urgency of the situation.

## Data Availability

The data that support the findings of this study are available from the corresponding author upon reasonable request.

## Abbreviations

CT: Computed tomography
NCD: National Clinical Database

## References

[1] Takahashi R, Fujikawa T (2021). Impact of perioperative aspirin continuation on bleeding complications in laparoscopic colorectal cancer surgery: a propensity score-matched analysis. Surg Endosc.

[2] Malik AH, East JE, Buchanan GN, Kennedy RH (2008). Endoscopic haemostasis of staple-line haemorrhage following colorectal resection. Colorectal Dis.

[3] Hébert J, Eltonsy S, Gaudet J, Jose C (2019). Incidence and risk factors for anastomotic bleeding in lower gastrointestinal surgery. BMC Res Notes.

[4] Kakeji Y, Takahashi A, Hasegawa H, Ueno H, Eguchi S, Endo I (2020). Surgical outcomes in gastroenterological surgery in Japan: Report of the National Clinical Database 2011–2018. Ann Gastroenterol Surg.

[5] Freund MR, Kent I, Horesh N, Smith T, Emile SH, Wexner SD (2022). Pancreatic injuries following laparoscopic splenic flexure mobilization. Int J Colorectal Dis.

[6] Issa IA, Soubra O, Nakkash H, Soubra L (2012). Variables associated with stress ulcer prophylaxis misuse: a retrospective analysis. Dig Dis Sci.

[7] Alhazzani W, Alshahrani M, Moayyedi P, Jaeschke R (2012). Stress ulcer prophylaxis in critically ill patients: review of the evidence. Pol Arch Med Wewn.

[8] Ueda T, Mori H, Sekiguchi T, Mishima Y, Sano M, Teramura E (2022). Successful endoscopic hemostasis compared to transarterial embolization in patients with colonic diverticular bleeding. J Clin Biochem Nutr.

[9] Sharma S, Prasad R, Gupta A, Dwivedi P, Mohindra S, Yadav RR (2020). Aneurysms of pancreaticoduodenal arcade: Clinical profile and endovascular strategies. JGH Open Access J Gastroenterol Hepatol.

[10] Tan EWK, Shelat VG, Monteiro AY, Low JK. Spontaneous retroperitoneal haemorrhage from pancreatoduodenal artery (PDA) rupture and associated complications. BMJ Case Rep. 2022;15(10). 10.1136/bcr-2022-250383 PMC9577903 36253011

[11] Casey L, Gananadha S, Jones A (2022). Ruptured pancreaticoduodenal artery aneurysm with median arcuate ligament compression: a two staged approach to management. EJVES Vasc Forum.

[12] Stanley JC, Wakefield TW, Graham LM (1986). Clinical importance and management of splanchnic aneurysm. J Vasc Surg.

